# Alcohol and Cannabis Use and the Developing Brain

**DOI:** 10.35946/arcr.v41.1.11

**Published:** 2021-09-09

**Authors:** Briana Lees, Jennifer Debenham, Lindsay M. Squeglia

**Affiliations:** 1 Matilda Centre for Research in Mental Health and Substance Use, University of Sydney, Camperdown, Australia; 2 Department of Psychiatry and Behavioral Sciences, Medical University of South Carolina, Charleston, South Carolina

**Keywords:** alcohol, cannabis, adolescence, brain, cognition, neuroimaging

## Abstract

**PURPOSE:**

Alcohol and cannabis are the most commonly used substances during adolescence and are typically initiated during this sensitive neurodevelopmental period. The aim of this review is to provide a comprehensive overview of the most recent literature focused on understanding how these substances affect the developing brain.

**SEARCH METHODS:**

Articles included in this review were identified by entering 30 search terms focused on substance use, adolescence, and neurodevelopment into MEDLINE, Embase, PsycINFO, ProQuest Central, and Web of Science. Studies were eligible for inclusion if they longitudinally examined the effect of adolescent alcohol and/or cannabis use on structural or functional outcomes in 50 or more participants.

**SEARCH RESULTS:**

More than 700 articles were captured by the search, and 43 longitudinal studies met inclusion criteria, including 18 studies focused on alcohol use, 13 on cannabis use, and 12 on alcohol and cannabis co-use.

**DISCUSSION AND CONCLUSIONS:**

Existing studies suggest heavy alcohol and cannabis use during adolescence are related to small to moderate disruptions in brain structure and function, as well as neurocognitive impairment. The effects of alcohol use include widespread decreases in gray matter volume and cortical thickness across time; slowed white matter growth and poorer integrity; disrupted network efficiency; and poorer impulse and attentional control, learning, memory, visuospatial processing, and psychomotor speed. The severity of some effects is dependent on dose. Heavy to very heavy cannabis use is associated with decreased subcortical volume and increased frontoparietal cortical thickness, disrupted functional development, and decreased executive functioning and IQ compared to non-using controls. Overall, co-use findings suggest more pronounced effects related to alcohol use than to cannabis use. Several limitations exist in the literature. Sample sizes are relatively small and demographically homogenous, with significant heterogeneity in substance use patterns and methodologies across studies. More research is needed to clarify how substance dosing and interactions between substances, as well as sociodemographic and environmental factors, affect outcomes. Larger longitudinal studies, already underway, will help clarify the relationship between brain development and substance use.

Adolescence is marked by significant social, emotional, cognitive, and physical changes, as individuals transition from childhood to adulthood. Although the exact definition of adolescence tends to vary, recent findings regarding adolescent development and growth include individuals between the ages of 10 and 24.^1^ Consistent with this defined age range, the human brain continues to develop until approximately age 25.^2^–^4^ Overall, total brain volume does not change during adolescence; however, there are significant microstructural changes in gray and white matter volume. Specifically, development of gray matter (i.e., neuronal cell bodies, dendrites) follows an inverted U-shaped curve, whereby volume increases until approximately ages 12 to 14, followed by a gray matter decrease due to synaptic pruning, changes in the extracellular matrix, and white matter encroachment.^5^–^7^ In contrast, white matter, which consists of neuronal axon tracts that connect gray matter regions, develops linearly into the mid-20s, as neural connections are optimized.^2^,^8^ Together, these structural changes in gray and white matter between ages 10 and 24 are related to significant socioemotional and cognitive development. Most prominently, emotion and reward-related regions of the brain mature fully during adolescence, while higher-order cognitive functions such as cognitive control, decision-making, planning, and working memory are slower to develop.^2^ These neural changes are believed to lead to heightened sensation seeking, impulsivity, and reward responsiveness during adolescence, as well as reduced ability to inhibit emotions and behaviors.^9^,^10^ This imbalance between reward and cognitive control also is believed to contribute to greater risk taking, including the initiation and escalation of substance use.^11^ These neural changes leave youth more vulnerable to the potentially serious and long-lasting consequences of substance use.^12^,^13^

Emerging research supports the notion that substance use disorders are developmental problems that begin during adolescence and have negative consequences on individuals throughout the life span.^14^,^15^ Alcohol and cannabis are the most commonly used substances during adolescence and are typically initiated during this important neurodevelopmental period, with patterns of use ranging from low and infrequent to heavy and problematic.^16^ Globally, alcohol is the most commonly used substance with 27% of 15- to 19-year-olds reporting alcohol use in the past month, with rates peaking to 41% for 20- to 24-year-olds. 17 Early alcohol use is related to poorer long-term outcomes; the prevalence of lifetime alcohol use disorder is 41% for those initiating alcohol use by age 12, compared to 17% and 11% for those initiating use at ages 18 and 21, respectively.^18^ Cannabis is the second most commonly used substance during adolescence, with overall rates of use increasing globally, particularly in regard to rates of daily use.^19^ Past-year cannabis use among 15- to 16-year-olds is highest in the Oceania region (18%), the Americas (12%), and Europe (12%), with rates of use increasing and peaking in 20- to 24-year-olds.^19^

Given the high rates of alcohol and cannabis use during adolescence, coupled with the significant neural maturation occurring during this period, it is critical to understand how alcohol and cannabis use affect adolescent brain development. Although other reviews exist on these topics, they have limitations. Specifically, existing reviews exclusively focus on alcohol,^12^,^20^ cannabis,^21^ or co-use,^22^ with some focusing solely on neuropsychological^23^ or neuroimaging studies^24^–^27^ within each substance use group. The aim of this review is to provide a comprehensive overview of the most recent literature that is both (1) focused on alcohol, cannabis, and alcohol and cannabis co-use use during adolescence and (2) meets the criteria for a prospective longitudinal neuropsychological and neuroimaging study in humans. Limitations of existing studies and future directions for research are discussed.

## Search Methods

Articles included in this review were identified via literature searches using MEDLINE, Embase, PsycINFO, ProQuest Central, and Web of Science, conducted on February 19, 2021. To capture the effects of alcohol and/or cannabis use on neural and cognitive development during adolescence, search terms included: (1) alcohol, cannabis, marijuana; (2) adolescen*, teenage*, young people, youth, emerging adult, young adult, college student; and (3) neuroimag*, neuroscience, PET scan, brain imag*, spectroscop*, magnetic resonance imag*, fMRI, sMRI, magnetic resonance spectroscopy, electroencephalogram, diffusion tensor imag*, structural imag*, functiona imag*, neuropsychological test, cogniti*, verbal working memory, episodic memory, visuospatial working memory, verbal fluency test, executive function*. In keeping with previous reviews,^12^,^28^ studies were eligible for inclusion in this narrative review if they met the following criteria: (1) examination of the effect of alcohol and/or cannabis use on neurodevelopment, including brain structure, brain function, and neuropsychological function; (2) longitudinal study with two or more neuroimaging or neuropsychological assessments; (3) adolescent sample ages 10 to 25 at baseline; and (4) sample size of 50 or more participants to reduce the likelihood of spurious findings. Cross-sectional studies are not included.

## Results

### Overview

**Figure 1:**
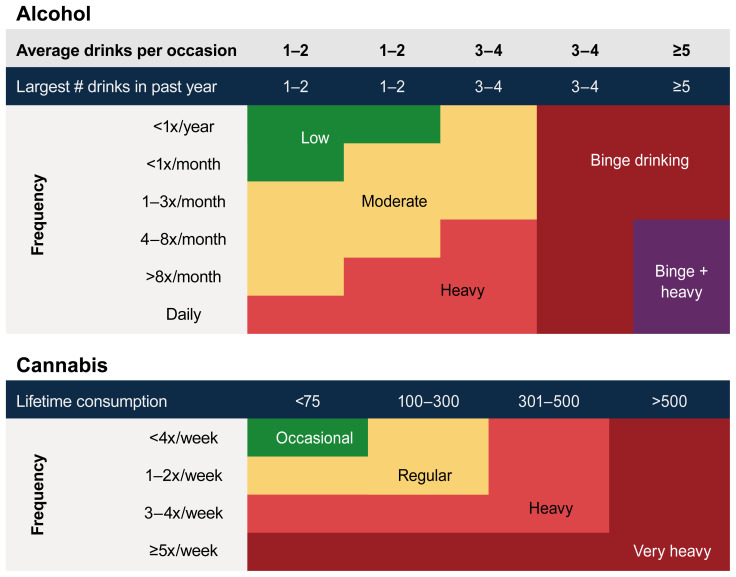
Typology of alcohol and cannabis use during adolescence. The charts are based on existing data classifying substance use groups during adolescence. Cannabis consumption is measured in occasions of cannabis use.^12^,^28^

More than 700 articles were captured by the search; and 43 longitudinal studies met inclusion criteria, including 18 studies focused on alcohol use, 13 on cannabis use, and 12 on alcohol and cannabis co-use. The effects of alcohol and cannabis use on ongoing adolescent neurodevelopment are described, portioned by brain structure (i.e., macrostructural and microstructural effects), brain function (i.e., resting state connectivity, task-based neural response), and neuropsychological effects (i.e., executive functions, impulsivity, attention, learning and memory, visual processing, verbal ability, psychomotor speed, IQ). Information on levels and typologies of alcohol and cannabis use (see Figure 1), age, and race/ethnicity details are described where available. To enable comparison across studies, the terms used in each study to describe the level of substance use (i.e., heavy drinking) have been standardized to align with the figure.

Where applicable, sex-specific findings are reported. Studies focused on alcohol effects are summarized first, followed by cannabis, then co-use studies. Two consortium-sized studies have examined the effect of substance use on the developing brain, including IMAGEN^29^ and the National Consortium on Alcohol and Neurodevelopment in Adolescence (NCANDA).^30^ IMAGEN is a multicenter brain imaging study of 2,216 adolescents recruited at age 14 from eight sites in Germany, the United Kingdom, and France. At baseline, 53% of youth reported lifetime alcohol use, 30% had smoked tobacco, and 12% had tried another substance, including 7% who had tried cannabis. NCANDA is a prospective longitudinal study being conducted in the United States across five sites, following 831 youth ages 12 to 21 who were required to have had limited exposure to alcohol at baseline (i.e., ≤ 5 drinking days for youth ages 12 to 15, ≤ 11 drinking days for youth age 16, ≤ 23 drinking days for youth age 17, ≤ 51 drinking days for youth age ≥ 18) or other substances (i.e., ≤ 5 days with cannabis use for youth age 12, with an additional five uses allowed per 1-year increase in age).^30^ A number of studies described below utilize subsamples from these data sets.

### Macrostructural Effects on Brain Structure

A number of longitudinal structural magnetic resonance imaging (MRI) studies have explored changes in brain volume and cortical thickness that occur across time following alcohol or cannabis use during adolescence. Several studies have delineated the post-substance use effects on brain structure by comparing youth who have and have not consumed alcohol or cannabis, and some have explored the relationship between levels of use and structural effects.

#### Alcohol

Among 483 NCANDA participants (baseline mean age = 16; 73% White, 14% Black, 11% Asian, and 2% undisclosed race/ethnicity), a portion of youth initiated moderate (13%) or heavy (13%) drinking by the 2-year follow-up assessment.^31^ Youth who drank heavily (see Figure 1) exhibited accelerated decreases in frontal gray matter volume in a dose-dependent manner when compared to controls, who drank little or not at all. Importantly, no significant group differences in frontal brain volume were observed at baseline prior to drinking onset, suggesting that aberrant volumetric trajectories were the result of alcohol uptake. By the 3- to 4-year follow-up assessment (*n* = 548), 22% of youth were drinking moderately and 18% were drinking heavily.^32^ Both moderate and heavy drinkers continued to exhibit altered neurodevelopmental trajectories with a graded dose effect, including accelerated cerebellar gray matter decline, white matter expansion, and cerebrospinal fluid volume expansion relative to controls. Notably, the authors did not explore baseline group differences prior to the onset of alcohol use; thus, pre-existing volumetric differences may be contributing to the observed effects. Interestingly, occasional cannabis co-use did not contribute to the effects in either study.^31^,^32^

The alcohol effects observed in the NCANDA sample are consistent with three smaller longitudinal studies of adolescents with a mean age of 15 to 18 at baseline (*N =* 55 to *N* = 134; 64% to 95% of the samples were White).^33^,^34^ These studies demonstrated that heavy drinking over 2 to 4 years was associated with accelerated decreases in frontal, parietal, and temporal gray matter volume and frontal cortical thickness.^33^,^34^ Additionally, these studies have reported attenuated increases in white matter growth over time for people who drank heavily when compared to the control group, who did not drink.^33^,^34^ One of these studies observed no group differences in cortical thickness or white matter volume at baseline, indicating that the effects were the result of alcohol consumption.^34^ However, a follow-up study found that pre-existing differences may partially contribute to neural outcomes among individuals who initiate alcohol use,^35^ consistent with previous reviews.^11^ Occasional cannabis use (mean days of cannabis use over 3 months before scan = 5) did not contribute to the observed effects in one study.^33^

In contrast to the studies above, findings from the IMAGEN study suggest that drunkenness frequency in 726 participants (100% White) was not associated with gray matter volume between ages 14 and 19 when controlling for sociodemographic, puberty, and substance-related confounding factors.^36^ Interestingly, a directionality analysis demonstrated that aberrant development of gray matter in the frontal and temporal regions prior to alcohol use was associated with increased prospective drunkenness frequency throughout adolescence. The discrepancy in findings between studies may relate to the age of participants as well as to differences in pre-existing factors. Robert et al. examined the effects of alcohol use during early adolescence (age 14) when youth had been consuming alcohol for a relatively short period of time,^36^ whereas the other studies reviewed here examined the effect during late adolescence (ages 17 to 21) where youth typically exhibited a longer drinking history. Therefore, macrostructural disruption may be a function of greater cumulative alcohol use across adolescence.

#### Cannabis

A study focused on the effect of adolescent cannabis use in the IMAGEN cohort (*n* = 706) indicated that greater consumption (i.e., occasional to regular use, see Figure 1) between ages 14 and 19 was associated with reduced expansion of the hippocampus and parahippocampus.^37^

#### Alcohol and cannabis co-use

Examination of adolescents engaging in heavy and frequent cannabis and alcohol co-use (mean lifetime days of cannabis use = 1,110; mean lifetime days of alcohol use = 605) found that heavy co-use was associated with a global reduction in cortical thinning (i.e., increased thickness) compared to controls, with frontal and parietal lobes being most consistently affected.^38^ Given that heavy alcohol use has been associated with decreased cortical thickness, heavy cannabis use may result in a differentiated pattern of macrostructural disruptions throughout late adolescence. Furthermore, one study examined the effect of monitored abstinence from heavy alcohol and cannabis use on macrostructural recovery among youth (*N* = 54; 76% White) who initiated use during middle adolescence.^39^ Participants who engaged in heavy alcohol and cannabis use throughout middle and late adolescence continued to exhibit thicker cortices than controls following 4 weeks of monitored abstinence, consistent with the co-use study described above.^38^ Further research is required to determine whether the deleterious effects of substance use on macrostructural development recede following reductions in use.

### Microstructural Effects on Brain Structure

Diffusion tensor imaging studies measure the microstructural integrity of white matter by mapping the diffusion pattern of water molecules.^40^ Common diffusion tensor imaging metrics include fractional anisotropy (FA), a measure of diffusion anisotropy or the unidirectionality of diffusion within a voxel; mean diffusivity (MD), a measure of diffusion magnitude; and axial diffusivity, a measure of the magnitude of diffusion parallel to the primary direction of diffusion, which may be a marker of axonal damage. White matter integrity following alcohol and cannabis use was examined in six studies, including four alcohol use studies and two alcohol and cannabis co-use studies.

#### Alcohol

In a study utilizing 4 years of NCANDA data, a whole-brain FA analysis of 451 adolescents was conducted.^41^ Youth who drank heavily (89% White) exhibited greater widespread FA reductions compared to no- and low-drinking controls, with a dose-dependent response observed. Interestingly, alcohol-associated disruptions were greater among youth ages 14 to 19.3 compared to youth ages 19.4 to 25 and were most pronounced in the genu and body of the corpus callosum, regions known to continue to develop throughout adolescence.^42^ Here, FA trajectories over 4 years were not correlated with occasional to regular cannabis use. Similarly, a 2-year study of 55 adolescents (95% White) observed relative FA decreases in temporal and subcortical regions among youth who initiated regular alcohol use between ages 17 and 18 relative to non-using controls.^34^ Sex differences were observed in one study. In a sample of 113 adolescents who were alcohol-naïve and ages 12 to 16 at baseline, greater alcohol consumption over the 3-year follow-up period was associated with greater FA reductions and mean diffusivity increases in the splenium of the corpus callosum and posterior thalamic radiation among males, and the opposite direction of effects was observed among females.^43^ Interestingly, sex hormones partially explained the effect of alcohol use on white matter microstructure.

#### Alcohol and cannabis co-use

Two studies from the same research group assessed youth who reported alcohol and cannabis co-use, and together they found limited evidence to suggest cannabis is neurotoxic to white matter integrity.^44^,^45^ The first study investigated the effect of continuing heavy cannabis and alcohol use (and occasional other substance use) over 18 months among 92 adolescents ages 16 to 21 at baseline (58% White).^44^ Greater alcohol use over the follow-up period was related to higher mean diffusivity bilaterally in the superior longitudinal fasciculus and higher axial diffusivity in the left posterior corona radiata; cannabis use was not correlated with diffusion indices. The second study examined the same cohort over 3 years (*N* = 54, 74% White) and compared older adolescents engaging in concurrent binge drinking and heavy cannabis use to those engaging in binge drinking only or no substance use.^45^ Youth in both the binge drinking only and co-use groups exhibited similar widespread FA reductions, with no added deleterious effect observed in the co-use group. Thus, the evidence to date indicates that heavy alcohol use and binge drinking, but not cannabis use, result in neurotoxic microstructural effects among younger and older adolescents.

#### Summary

Heavy alcohol use during late adolescence is associated with accelerated widespread decreases in gray matter volume and cortical thickness throughout frontal, parietal, temporal, and cerebellar regions. Additionally, attenuated white matter growth and poorer white matter integrity throughout widespread regions have been observed among heavy drinkers, with greater disruptions from consumption during middle to late adolescence than during young adulthood.

Heavy cannabis use may be associated with a differentiated neural pattern than alcohol use alone. Cannabis use is associated with macrostructural consequences only, including reduced expansion of the hippocampal region and increased cortical thickness in the frontal and parietal lobes. Early evidence suggests that disruptions in macrostructural development due to cannabis use do not recede over the short term; however, further research on the recoverability of substance-related macrostructural effects is required. New evidence also highlights the importance of studying sex and sex hormones when investigating the effects of alcohol and cannabis use on the adolescent brain;^43^ however, further research is required.

### Effects on Brain Function

Longitudinal studies have measured resting-state functional connectivity and neural response to cognitive tasks across time to examine the effect of alcohol and cannabis use on brain function.

#### Alcohol

Limited evidence is currently available on the specific effects of alcohol use on functional neurodevelopment. A 4-year study examined the effect of low-level alcohol consumption during middle to late adolescence on neural response to cognitive control tasks (*N* = 92).^46^ Low-level consumption (< one standard drink [14 grams of alcohol] per week at age 14 to < four drinks per week by age 18) did not impair ongoing maturation of cognitive control networks, with similar increases in activation of the anterior cingulate cortex and pre-supplementary motor area over time among non-drinkers and low-level drinkers. Meanwhile, the effect of heavy alcohol consumption during adolescence was examined in a study utilizing three annual assessments of resting-stage functional MRI data from the NCANDA cohort (*N* = 526).^47^ To explicate the specific effects of alcohol use, any clusters correlated with cannabis use were omitted from the analysis. Higher levels of alcohol consumption over the follow-up period were related to greater within-network connectivity in two motor networks, and these effects were mediated by sensation seeking. Interestingly, alcohol use effects were more pronounced in female adolescents than in male adolescents, with a graded dose effect observed.

#### Cannabis

The effect of prolonged and heavy cannabis use on functional neurodevelopment was examined in four studies. Leveraging the IMAGEN data set, prolonged occasional to regular cannabis use from ages 14 to 19 was associated with relative declines in neural reactivity to angry faces across time when compared to substance-naïve matched controls (*n* = 76).^48^ Of note, this effect was no longer significant when the participants who used cannabis were compared to the larger, unmatched sample of naïve participants (*n* = 502). In a younger sample of adolescents ages 12 to 15 (*N* = 67, 67% White), the initiation of occasional cannabis use was associated with decreased activity in the cuneus during visuospatial working memory compared to controls; however, there were no changes to cognitive scores.^49^ In a study of 65 youth ages 10 to 23 at baseline (mean age = 17), adolescents engaging in very heavy cannabis use and seeking treatment for cannabis use disorder exhibited a decline in resting functional connectivity between the anterior cingulate cortex and the dorsolateral and orbitofrontal cortices across 18 months.^50^ Finally, one study investigated neurofunctional recovery following 4 weeks of monitored abstinence from cannabis after 6 years of very heavy cannabis use (average joints per year = 899; total lifetime days of use = 5,268).^51^ Abstinence was associated with a reduction in the magnitude of functional differences between the cannabis use and control groups. Compared to controls, abstinent cannabis users showed a differentiated pattern of connectivity within the insula and default mode networks as well as stronger anticorrelation between them. A graded dose effect was observed, where the extent of persistent alterations in functional activity was related to the amount of cannabis previously used.^51^

#### Alcohol, cannabis, and tobacco co-use

The effect of any substance use—including alcohol, cannabis, and tobacco—on adolescent brain function was assessed annually across 4 years among 167 adolescents ages 13 to 14 at baseline.^52^ Greater substance use over time was related to increased insula activation during risk processing, with more pronounced effects for adolescents with low compared to high cognitive control. This study did not explore independent effects of alcohol and cannabis use on neural activation.

#### Summary

Overall, preliminary evidence indicates that heavy alcohol use during adolescence disrupts the maturation of network efficiency in a dose-dependent manner, with more significant effects observed among females. Even relatively low-level cannabis use (i.e., occasional and regular consumption) as well as heavier use during adolescence may alter the rate of neurotypical functional development in brain regions important for cognitive control. Some neural recovery may be possible after abstinence; however, months or years may be required for complete recovery of functional connectivity from heavy cannabis use. Preliminary evidence underscores that cognitive control and sensation-seeking behaviors could be an important target in prevention and treatment of substance use in adolescents, given the moderating roles on neurofunctional effects. Further research is required to determine the relative effects of alcohol and cannabis consumption on functional neurodevelopment and whether neural recovery occurs following reductions in use.

### Effects on Neuropsychological Function

Neuropsychological tests enable tracking of cognitive skills over time to uncover the effect of alcohol and cannabis use on cognitive development. The following sections summarize the reported effects by neuropsychological domain.

#### Executive functions

Executive functions refer to a range of top-down mental processes that enable an individual to hold concentration and attention. There are three core executive functions, including inhibition, working memory, and cognitive flexibility.^53^,^54^ From these core functions, other higher-order executive functions are built, such as reasoning, problem-solving, decision-making, and planning.^55^ Mediated by frontal lobe development, these functions are essential for educational and occupational success, mental and physical health, and social development.^55^ Current evidence suggests that alcohol consumption during adolescence does not impair maturation of executive functions. A 4-year study of 92 adolescents found that greater cumulative low-level alcohol consumption (< four drinks per week at age 18) between ages 14 and 18 did not have an effect on conflict monitoring or updating of working memory performance and was associated with subtle improvements in inhibitory control.^46^ Likewise, a study using data from 2,226 adolescents in the Tracking Adolescents’ Individual Lives Survey (TRAILS) found that 4 years of occasional or frequent low or heavy alcohol use was not associated with deterioration in inhibition, working memory, or cognitive flexibility, compared to no alcohol use.^56^,^57^ In this study, cannabis use at ages 16 and 19 was not correlated with executive functioning performance across ages 11 to 19. Lastly, a 4-year study of 234 adolescents unexpectedly found that more alcohol use predicted better working memory, driven largely by a positive relationship between recent blackout history and auditory attention scores, when controlling for sociodemographic factors.^58^ Notably, no follow-up tests supported the unexpected working memory finding, such as removing sex and other covariates from the regression models.

Similarly, most studies examining young adults have not found detrimental effects of alcohol use on executive functioning development. A 4-year study followed 155 young adults every 22 months from age 18. Individuals who reported consistent binge drinking throughout the entire study showed no disadvantage for decision-making ability when compared to non–binge-drinking controls, who consumed four drinks per week on average.^57^ Here, occasional cannabis use was not related to decision-making ability. Meanwhile, an assessment of 436 Dutch young adults (mean age = 21 years) showed baseline alcohol use of any level (i.e., abstinence, occasional moderate, frequent moderate, occasional heavy, frequent heavy) was not related to planning or reasoning ability 11 months later, nor was change in average alcohol consumption over time.^59^ Finally, another study assessed 89 young adults ages 18 to 20.^60^ Compared to non–binge-drinking controls, individuals who reported consistent binge drinking over 2 years exhibited poorer conflict monitoring at both time points. However, consistent binge drinking over 2 years was not associated with deterioration in working memory or planning across time. Additionally, occasional cannabis use was not associated with performance.

As described above, studies that focused on the impact of alcohol use have not reported an effect of occasional cannabis use on executive functioning maturation throughout adolescence and young adulthood.^56^,^57^,^60^ Meanwhile, the Co-Venture study assessed 3,826 adolescents with a mean age of 13 at baseline who were assessed annually for 5 years.^61^ Cannabis use ranged in frequency from occasional to very heavy use (i.e., daily); and when accounting for alcohol use, the female cannabis users were shown to be more sensitive to negative consequences of working memory than were the males. Data from the Dunedin Study of 1,037 individuals showed that adolescent-onset and persistent very heavy cannabis use was associated with impaired working memory and perceptual reasoning over more than 20 years.^62^ Another study assessed 175 adolescents ages 12 to 15 at baseline across the course of 14 years.^63^ Greater cumulative cannabis use over adolescence was associated with poorer inhibitory control. Finally, a study assessing the effect of cannabis use among 58 young adults age 19 over a 2-year period (82% White) found that cannabis consumption declined from very heavy to heavy, which corresponded to improvements in working memory, planning, and motivated decision-making, suggesting that deficits may be associated with very heavy use only and that these higher-order cognitive functions are recoverable following reductions in consumption.^64^

Overall, there is no strong, consistent evidence to indicate that low to heavy alcohol use during adolescence or young adulthood disrupts executive functioning maturation across time. Longitudinal data on cannabis use and executive functioning performance suggest that frequent consumption and greater cumulative use across adolescence may disrupt inhibitory control, working memory (particularly in females), planning, and decision making.

#### Impulsivity

Impulsivity is defined as a behavior characterized by little or no forethought, reflection, or consideration of consequences, when compared to actions by individuals with similar skill and knowledge levels. Impulsivity is thought to be related to risk-taking behaviors.

Two studies examined the impact of alcohol use on impulse control across adolescence; however, no studies have examined the impact of cannabis use or co-use of these substances. IMAGEN data from 304 young people ages 13 to 14 at baseline found that over a 2-year period, adolescents who reported more than 40 occasions of alcohol use exhibited increases in trait impulsivity, while youth who reported alcohol use on fewer than 10 occasions exhibited decreases in impulsivity.^65^ Likewise, a study of 116 adolescents with an average age of 14 at baseline demonstrated that greater total lifetime drinks over approximately 2 years predict escalated impulsive choice across time.^66^ In both studies, limited cannabis use was reported. Therefore, the transition into frequent drinking in early to middle adolescence may disrupt normative developments in impulse control.

#### Attention

Attentional control has been measured in two longitudinal studies focused on the effects of low to heavy alcohol use; in two studies focused on effects of heavy cannabis use; and in three studies exploring co-use of alcohol and cannabis.

The TRAILS study of 2,226 adolescents reported that 4 years of weekly low or heavy alcohol use did not have an effect on sustained attention, when compared to controls who consumed no alcohol.^56^ However, sex differences were identified in a 5-year study of 89 adolescents age 14 at baseline (76% White), where more hangover symptoms (from heavy alcohol use) in the previous year predicted relative worsening of sustained attention in males only.^67^ Heavy cannabis use did not predict change in attention across time in this study.

In terms of cannabis-related effects, declines in cannabis use from very heavy to heavy consumption correspond with improvements in attention.^64^ Likewise, a study of 74 youth ages 16 to 26 (66% White) found that 2 weeks of monitored abstinence from very heavy cannabis use was associated with improvement in attention compared to controls.^68^ Together, these data suggest that very heavy cannabis use during adolescence and young adulthood is associated with diminished attention; however, such deficits may recover following reductions in use.

Additionally, alcohol and cannabis co-use has been associated with progressive declines in attentional control across time. In a study of 69 adolescents (80% White) observed from ages 13 to 19, the initiation of concurrent use was related to deficits in complex attention compared to substance-naïve counterparts.^35^ A negative dose-response relationship also has been observed over an 8-year period from ages 16 to 24 (78% White), where greater co-use of cannabis and alcohol among 73 adolescents was related to poorer attention.^69^ Interestingly, when assessing the relative effects of concurrent heavy alcohol and cannabis use over 3 years among 108 adolescents (63% White), attentional differences appeared to be driven by alcohol rather than cannabis use.^38^

In summary, previous studies have identified attentional deficits among heavy drinking males and heavy cannabis users. Initiation of co-use of these substances in adolescence has predicted poorer attention, with graded dose effects observed that may be driven by alcohol use. Early evidence suggests that adolescents may recover from cannabis-related effects following reductions in use. Recoverability from alcohol effects remains unknown.

#### Learning and memory

Inextricably linked to adolescent learning and memory development is educational attainment, one of the most critical developmental tasks for youth. Thus, substance-induced deficits are arguably even more impactful for young people than adults. Ten studies included in this review examined the effect of alcohol or cannabis use on learning and memory performance throughout adolescence.

Alcohol-focused studies have predominantly reported on the impact of heavy binge drinking. A 6-year study of 112 substance-naïve adolescents (mean baseline age = 13; 69% White) found that higher estimated peak blood alcohol concentration over the 3-month period before the follow-up neuroimaging session predicted worse verbal learning and immediate, short- and long-term delayed, and cued recall across time in a dose-dependent manner.^70^ Furthermore, a 6-year study following 155 older adolescents every 22 months from age 18 found that consistent binge drinking was associated with deficits in immediate and delayed recall, with similar deficits for males and females when compared to non–binge-drinking controls.^71^ Occasional cannabis use did not influence the effects. Similarly, previously described studies assessing the impact of the frequency of drinking days in middle adolescence^58^ and consistent binge drinking in late adolescence^60^ have observed poorer performance on immediate and delayed recall as well as on retention after 2 to 4 years of continued use. In contrast to these findings, one study reported that occasional or frequent alcohol use at moderate or heavy levels was not related to short-term delayed recall performance 11 months later among young adults with a mean age of 21.^59^ However, study authors note that the null findings should be interpreted with caution given the high variance in cognitive performance. Two studies have focused on the effect of adolescent cannabis use on learning and memory performance. One study examined the impact of early (< age 16) and late (≥ age 16) onset of cannabis use on learning ability among 119 young people (89% Black).^72^ On one of four tests, early-onset cannabis use was associated with a small decline in structured learning performance compared to no use; however, neither group exhibited suboptimal learning trajectories on the majority of tests. Additionally, in a large representative cohort of young adults ages 20 to 24 at baseline (*n* = 1,978), occasional cannabis use was associated with decreased immediate recall compared to young people with long-term abstinence from cannabis use, suggesting recovery may be possible after long-term abstinence.^73^

Alcohol and cannabis co-use has been shown to impair learning and memory, with preliminary evidence implicating alcohol as the predominant driver of these effects. The effect of heavy alcohol and cannabis use (where participants met criteria for alcohol use disorder and engaged in other substance use) on learning and memory trajectories across 10 years was examined during middle to late adolescence.^74^ Examining 213 participants, heavier use patterns and greater hangover and withdrawal symptoms over time were related to poorer verbal learning and memory, suggesting a dose-dependent relationship between substance use and cognitive functioning. Similarly, a second study showed that adolescents with a history of substance use disorder (concurrent alcohol, cannabis, and stimulant use) demonstrated impairments in verbal learning and memory compared to youth without substance use disorder, when followed up seven times from ages 16 to 24 (*N* = 73, 78% White).^69^ Finally, a previously described study showed that adolescent engagement in concurrent heavy cannabis use and binge drinking over 18 months was associated with progressive declines in delayed recall when compared to those engaging in occasional cannabis use alone.^38^ Further analysis of this cohort at the 3-year follow-up where groups reported congruent levels of alcohol use suggested that the memory deficits may be a result of alcohol rather than cannabis use.

Overall, studies focused on alcohol use during adolescence have observed a disruption in learning and memory development following heavy and binge drinking, with the severity of effects related to levels of consumption. Occasional cannabis use has been shown to have a negative effect on recall but not on learning. Meanwhile, heavy co-use for up to 10 years is related to poor outcomes, which may be driven by the effects of alcohol use rather than cannabis use.

#### Visual processing

Visual processing involves the brain’s analysis and interpretation of visual signals. Seven previously described studies have examined the impact of alcohol and cannabis use on visual processing ability across adolescence, including four alcohol-focused studies and three co-use studies.

Initial evidence from the previously described study suggests that low-level alcohol use during adolescence does not have a negative effect on the development of rapid visual processing.^46^ In contrast, heavy alcohol use and withdrawal symptoms during middle to late adolescence have been associated with prospective declines in visuospatial function over 10 years, compared to controls.^75^ Additionally, a dose-dependent effect has been observed among 234 adolescents ages 12 to 14 at baseline, where greater number of drinking days over 4 years predicted visuospatial ability.^58^ Examination of sex differences suggests that this effect may be particularly strong among young females.^67^

Others studies have found that adolescent engagement in heavy cannabis use and binge drinking over 3 years has resulted in significant declines in visuospatial functioning, with effects driven by alcohol use.^38^ Moreover, greater cumulative cannabis use over 14 years and proximal increases in alcohol consumption predict decrements in visuospatial functioning.^63^ Notably, 4 weeks of monitored abstinence from concurrent cannabis use and binge drinking were not associated with improvements in visuospatial functioning.^39^ Overall, there is consistent evidence that heavy alcohol use during middle to late adolescence leads to poorer visual processing and functioning. Performance does not appear to improve over the short term following a period of abstinence.

#### Verbal ability

Verbal ability refers to the ability to both understand and communicate effectively with words. Comprehension and verbal fluency are considered parts of verbal ability.

Two large cohorts of twins (cohort 1, *n* = 2,277; cohort 2, *n* = 1,241) show that the initiation of occasional cannabis use was associated with a decline in verbal ability; however, this finding is not apparent in twins discordant for cannabis use (cohort 1, *n* = 94; cohort 2, *n* = 200).^76^ Additionally, persistent very heavy cannabis use over 20 years was predictive of impaired verbal comprehension (*n* = 1,037).^62^ No studies included in this review examined the effect of alcohol use or alcohol and cannabis co-use on verbal ability across adolescence.

#### Psychomotor speed

Psychomotor speed is defined as the relationship between cognitive and motor movements, often measured by both accuracy and speed. It includes movement, spatial relationships, and use of motor skills.

Preliminary evidence shows that alcohol and cannabis use in middle adolescence affects psychomotor development. Among 234 adolescents ages 12 to 14 at baseline, several substance use behaviors predicted psychomotor speed performance 4 years later.^58^ Specifically, more post-drinking effects from heavy-level alcohol use and greater substance use (including cannabis) was associated with slower psychomotor speed.

#### IQ

IQ is a standard measure of an individual’s intelligence level. Four studies included in this review examined the effect of occasional to very heavy cannabis use on IQ across adolescence. Two large cohorts of twins showed that the initiation of occasional cannabis use was associated with a decline in IQ; however, this finding was not apparent in twins discordant for cannabis use,^76^ suggesting IQ deficits may be attributable to confounding factors rather than the direct neurotoxic effect of cannabis. Similarly, another large study of twins (*N* = 1,989) demonstrated that the initiation of regular cannabis use was not associated with prospective IQ decline in discordant twins for cannabis use.^77^ The effect of heavier cannabis exposure on IQ was examined in a third study. When comparing 65 adolescents ages 17 to 20 who were current very heavy users (≥ 5 joints per week), current heavy users (< 5 joints per week), former users (no regular use for ≥ 3 months), and non-users, only the group with very heavy cannabis use showed any relative IQ decline across 8 years.^78^ Likewise, an additional study reported that adolescent-onset, persistent very heavy cannabis use over 20 years was associated with IQ declines across time (*N* = 1,037).^62^ No studies included in this review examined the effect of alcohol and cannabis co-use on IQ across adolescence.

#### Summary

A wealth of longitudinal studies have assessed the effect of adolescent alcohol and cannabis use on neuropsychological development. Based on the current evidence base, heavy alcohol use (including binge drinking) during adolescence disrupts normative developments in impulse and attentional control, learning and memory, visual processing and functioning, and psychomotor speed, with the severity of some effects dependent on dose. In contrast, low to heavy alcohol use during adolescence and young adulthood does not appear to disrupt executive functioning maturation across time. The recoverability of alcohol effects generally remains unknown.

Longitudinal data on cannabis use and neuropsychological development are generally lacking. Preliminary evidence suggests that heavy to very heavy use could lead to deteriorated development of executive functions and IQ. Heavy alcohol and cannabis co-use in adolescence has been linked to a range of deficits, including deficits in attentional control, learning and memory, visuospatial functioning, and psychomotor speed. The added effect of co-use versus singular use has not been adequately explored to date, although early evidence suggests that heavy alcohol use may be driving some of these effects.

## Discussion

The rapidly expanding literature of prospective, longitudinal studies tracking neurodevelopment and substance use has greatly increased knowledge of the effects of adolescent alcohol and cannabis use on brain structure, function, and cognition. Overall, it is clear that heavy alcohol use during adolescence is associated with neural and cognitive consequences (see Table 1). Although there is evidence to suggest that heavy cannabis use can affect ongoing neurodevelopment, early data from co-use studies indicate that alcohol could be partially driving these effects. Parsing out the interactive effects of alcohol, cannabis, and other substances is a key challenge in this field given that other substance use is often accompanied by alcohol use. Basic science and the large multisite human studies currently underway (i.e., IMAGEN, NCANDA, Adolescent Brain Cognitive Development [ABCD] Study) will help disentangle the neural and cognitive effects over the next decade. It is critical to differentiate substance-specific effects, especially given the growing legalization of cannabis use, the upsurge in adolescent vaping, and global concerns regarding opioid misuse.^79^,^80^ Further, understanding the recoverability from these effects following reductions in substance use is particularly important given the critical focus on continued educational attainment, learning, and ongoing neurodevelopment during adolescence.

An important observation from the current review is the need for more diverse samples. The vast majority of existing work has studied White youth from high socioeconomic backgrounds in the United States and Europe, limiting the generalizability of findings. Future studies also should improve racial descriptions of participants. Often studies report on the proportion of White versus non-White youth, with critical details of race and ethnicity representation overlooked. Another consideration likely reducing the generalizability of the current evidence base is the frequently reported eligibility criteria that excludes youth with co-occurring psychological and medical issues. Importantly, this has enabled specific examination of the effect of substance use on neurodevelopment; however, future studies should begin to explore the interactive effects of adolescent substance use and psychopathology on adolescent neurodevelopment. This knowledge will benefit practitioners working with adolescents and inform future initiatives on substance use prevention and mental health.

Overwhelmingly, the majority of studies thus far have examined effects related to low-level substance use initiation or heavy, frequent use. Although some studies report dose-dependent effects, greater clarification is needed to determine whether there is a threshold for harmful use that results in neural and cognitive consequences. The magnitude of neurodevelopmental consequences from alcohol and cannabis use is likely to stem from a multitude of other factors including sociodemographic characteristics, early-onset puberty, genetic polymorphisms, prenatal exposures, childhood adversity, and psychopathology, among other important factors, which may be lost in the standard mean group values used in analysis.^81^,^82^ Improved quantification of individual variation, as well as exploration of possible interactive effects and underlying mechanisms of neurodevelopmental consequences, are necessary to advance identification of youth who may be at risk for long-term negative effects.

Given ethical barriers surrounding adolescent substance use, this field of research is reliant on observational human studies, which creates challenges for establishing causality and directionality. This review aims to identify neurobiological and neuropsychological consequences of adolescent alcohol and cannabis use by summarizing prospective, longitudinal studies that repeatedly assess individuals over time as patterns of substance use emerge and escalate. However, many of the included studies used only two neuroimaging or neurocognitive time points, which does not allow for more complex modeling and understanding of developmental trajectories over time. Furthermore, reliably identifying causal mechanisms in observational studies without randomization is difficult, with the primary concern being confounding (i.e., whether causal associations are real, or entirely or partly confounded by other variables). The studies synthesized in this review included statistical models with a range of sociodemographic and environmental covariates to address the issue of confounding. However, numerous methods are now available in response to the confounding problem in observational data, such as Granger causal models, structural equation models, Bayesian networks, state-space models, regression discontinuity design, the difference-in-differences approach, and instrumental variable approaches.^83^ These techniques have the ability to improve causal understanding and should be utilized in future analyses of large-scale cohorts to delineate causal effects of alcohol and cannabis use.

An additional methodological concern identified in this review is the reliance on youth self-report of substance use. Several studies also used ranges in surveys to capture frequency and quantity of consumption, weakening the ability to explore graded dose effects. Utilization of real-time measures and biological markers can greatly increase the accuracy and reliably of substance use data.^84^,^85^ Although the reported studies focused on alcohol and cannabis use, polysubstance use (e.g., tobacco, cocaine, opioids) could affect findings. Although some studies controlled (or excluded participants) for co-occurring use of other substances, future studies with larger samples will be able to better understand the potential compounding effects of other substance use on brain development. Much of the data presented was collected before vaping existed; given the recent uptick in tobacco vaping, it will be important that future studies assess tobacco vaping to understand its unique effects on adolescent brain development. Furthermore, a greater selection of neuroimaging tools that track neurochemicals and transmitters in the brain (e.g., magnetic spectroscopy imaging, positron emission tomography) are now available. Understanding neurochemical changes could further improve understanding of the mechanisms underlying neural effects of substance use.

Cannabis potency has increased substantially over the past several decades.^86^ Quantifying cannabis use is a complex issue due to the lack of regulation and standardization in cannabis products.^87^ Most existing studies utilize crude measures of cannabis use (e.g., range of self-reported days of use over restricted periods of time), limiting the ability to understand dose-, time-, and potency-related relationships between cannabis use and neurodevelopmental outcomes. Notably, the National Institutes of Health has recently established a standard 5 mg delta-9-tetrahydrocannabinol (THC) unit to be used in research.^88^ Future studies should utilize this unit measurement and incorporate a more granular level of self-report data, as well as objective biomarkers of cannabis use, in an attempt to better understand how potency and quantity of use affects neurodevelopmental outcomes.^89^

## Conclusions

In summary, alcohol and cannabis are two of the most commonly used substances during adolescence, which is a critical developmental period associated with significant neurocognitive maturation. Longitudinal neuroimaging and neuropsychological research have helped clarify the effect of substance use on adolescent brain development. Existing studies suggest alcohol and cannabis use during adolescence are related to small to moderate disruptions in brain structure and function, as well as neurocognitive impairment (see Table 1). Overall, findings suggest more pronounced effects related to alcohol versus cannabis use; however, several limitations exist in the literature. Sample sizes are relatively small and demographically homogenous, with significant heterogeneity in substance use patterns and methodologies across studies. More research is needed to clarify how substance dosing and interactions between substances, as well as sociodemographic and environmental factors, affect outcomes. Larger longitudinal studies, already underway, will help clarify the relationship between brain development and substance use. Findings can be used to inform psychoeducational programming^90^,^91^ and provide important targets to developing substance use treatments for adolescents.^92^

## Figures and Tables

**Table 1:** Effects of Adolescent Alcohol and Cannabis Use on the Developing Brain

Size of Effect	Heavy Alcohol Use/Binge Drinking	Heavy Cannabis Use	Alcohol and Cannabis Co-Use
**Brain structure**			
Small to moderate	Disruptions observed in middle to late adolescence Widespread decreases in gray matter volume and cortical thickness Slowed white matter growth Poor white matter integrity, partially explained by differences in sex hormones	Decreases in subcortical volume Increases in frontoparietal cortical thickness Neurodevelopmental disruptions may not recover over the short term	
Small to large			No added deleterious effect of co-use on white matter integrity vs. alcohol use only
**Brain function**			
Small			Altered neural response in the insula during risk processing
Small to moderate	Disrupted maturation of network efficiency More significant effects among females		
Small to large		Altered rate of functional development in brain regions important for cognitive control Some neural recovery possible after abstinence	
**Neuropsychological function**			
Small to large	Disruptions in development of: ▪ Impulse and attentional control ▪ Learning and memory ▪ Visual processing and functioning, particularly in females ▪ Psychomotor speed	Disrupted executive functioning development, particularly in females Decreased IQ with very heavy use Improvements in working memory, planning, decision-making, and attention following reduced use	Attention deficits Poor psychomotor speed Progressive declines in learning, memory, and visuospatial functioning (driven by alcohol use) Short-term abstinence not associated with improved visuospatial functioning
